# MAGE-A3 regulates tumor stemness in gastric cancer through the PI3K/AKT pathway

**DOI:** 10.18632/aging.204373

**Published:** 2022-11-08

**Authors:** Qi-Ying Yu, Zhi-Wen Wang, Meng-Ying Zhou, Shang-Fu Li, Xing-Hua Liao

**Affiliations:** 1 Institute of Biology and Medicine, College of Life and Health Sciences, Wuhan University of Science and Technology, Hubei 430081, China; 2 Yueyang People’s Hospital, Yueyang Hospital Affiliated to Hunan Normal University Neoplasm Ward 1, Yueyang 414000, Hunan, China; 3 Yueyang Vocational and Technical College, Yueyang Key Laboratory of Chronic Noncommunicable Diseases, Yueyang 414000, Hunan, China

**Keywords:** mRNAsi, WGCNA, gastric cancer, MAGE-A3

## Abstract

Gastric cancer remains a malignant disease of the digestive tract with high mortality and morbidity worldwide. However, due to its complex pathological mechanisms and lack of effective clinical therapies, the survival rate of patients after receiving treatment is not satisfactory. A increasing number of studies have focused on cancer stem cells and their regulatory properties. In this study, we first constructed a co-expression network based on the WGCNA algorithm to identify modules with different degrees of association with tumor stemness indices. After selecting the most positively correlated modules of the stemness index, we performed a consensus clustering analysis on gastric cancer samples and constructed the co-expression network again. We then selected the modules of interest and applied univariate COX regression analysis to the genes in this module for preliminary screening. The results of the screening were then used in LASSO regression analysis to construct a risk prognostic model and subsequently a sixteen-gene model was obtained. Finally, after verifying the accuracy of the module and screening for risk genes, we identified MAGE-A3 as the final study subject. We then performed *in vivo* and *in vitro* experiments to verify its effect on tumor stemness and tumour proliferation. Our data supports that MAGE-A3 is a tumor stemness regulator and a potent prognostic biomarker which can help the prediction and treatment of gastric cancer patients.

## INTRODUCTION

Gastric cancer remains one of the malignant tumors with the highest morbidity and mortality rates worldwide. According to Global Cancer Statistics, as of 2020, a total of 1,089,103 new gastric cancer patients have been diagnosed worldwide, accounting for 5.6% of the total new cancer patients. There were 768,793 new deaths from gastric cancer, accounting for 7.7% of the overall mortality [1]. Despite many significant advances in therapeutic strategies over the past decade, such as immunotherapy, chemotherapy, and radiation therapy, therapeutic efficacy is not ideal and the survival rate of patients after treatment remains poor [2, 3]. The conventional surgical resection is often associated with the risk of metastasis and recurrence, as most patients were diagnosed at advanced stage [4]. Hence, it is more urgent and practical to investigate the molecular mechanism of gastric cancer and further develop effective prognostic factors.

Recently, many studies have focused on a specific class of tumor cells, namely cancer stem cells. They are involved in most processes of disease progression and heterogeneity of tumor [5]. Cancer stem cells own some characteristics as normal stem cells, such as self-renewal and ability to differentiate into other cells that consist of various parts of the tumor [6]. Besides, cancer stem cells also possess their own characteristics. They usually stay at a dormant state for a long time and are highly resistant to drugs and insensitive to external physical and chemical environments that are detrimental to the cells [7]. Accumulating evidence suggests that cancer stem cells take the main responsibility for post-surgical recurrence, tumor metastasis, resistance to chemotherapy and radiation therapy [8, 9]. Just for this reason, focusing on cancer stem cell therapy and exploring the key molecules that regulate the properties of tumor stem cells will greatly improve the likelihood of disease cure and patient survival rate.

To better investigate and characterize these molecules that regulate and maintain tumor stemness, Malta and his colleagues analyzed transcriptome and other profiles from the TGCA database to obtain an indices which could quantify stemness [10]. The mRNA expression-based stemness index (mRNAsi) is used to quantify the stemness of mRNA expression in samples, the epigenetic regulation based-index (EREG-mRNAsi) is utilized to characterize the effect of epigenetic modifications on stemness. By applying these tumor stemness indices, researchers can obtain molecules involved in the regulation of tumor stemness in different tumors in the TCGA database. Higher index scores represent more important in its regulation of tumor stemness. Therefore, we got these tumor stem cell indices and applied them to the present study.

In this study, we first identified tumor stemness-related modules and key genes by using the WGCNA and mRNAsi indices differentially expressed genes (DEGs). After extracting the expression data of these genes, we performed consensus clustering analysis on gastric cancer samples in TCGA. We found that gastric cancer patient samples could be classified into two tumor stemness subtypes (C1 and C2groups) based on these key genes. WGCNA were again applied to construct co-expression network and screen key genes after gastric cancer samples consensus clustering analysis. Then, we implemented an initial screening of the modules we were interested in and applied the LASSO regression analysis algorithm to construct a risk model and validated it. Finally, we identified MAGE-A3 as the final study subject. The results show that MAGE-A3 is involved in the regulation of tumor proliferation and that tumor stemness regulates through PI3K/AKT signalling pathways. Thus, our study provides a new potential target for the treatment and prognosis of gastric cancer.

## MATERIALS AND METHODS

### CCK-8 assay

To test the proliferative capacity of the cells, the CCK-8 (Thermo Fisher, USA) assay was performed. Inoculate 10,000 cells in wells of a ninety-six-well plate with three replicate wells per group. Continue all subsequent operations according to the kit instructions. The absorbance at 450 nm of each group was measured at 0, 24 and 72 hours after inoculation using a microplate reader.

### 5-ethynyl-2′-deoxyuridine (EdU) incorporation assay

Cells from the experimental and control groups were inoculated on cell coverslips at the same time. After overnight incubation at 37° C in 5% CO2, subsequent manipulations were performed as follows. Briefly, Replace the complete medium with fresh medium containing 20mM EDU (Thermo Fisher, USA) and incubated at 37° C for 2 hours. DAPI was used to stain cell nuclei. Olympus confocal microscope FV3000 was used to observe and take pictures.

### Immunofluorescence and confocal imaging

Cellular immunofluorescence is utilized to detect the expression of tumor stem cell biomarker protein levels. The experimental steps are briefly described as follows:1 Cells were inoculated on coverslips, cultured overnight and washed three times.2 Cells were fixed with 4% paraformaldehyde at room temperature and permeabilized with 0.1% Triton-100. 3 Sealing of antigens at room temperature 1 hour.4 Primary antibody (CD44, EpCAM; Abclonal, China) was incubated overnight at four degrees and CY3-labeled secondary antibody(Abclonal, China) was added. Olympus confocal microscopy (Olympus, Japan) was used for photography.

### Tumor xenograft model and animal imaging

Four-week-old immunodeficient nude mice, purchased from Beijing Huafukang Experimental Animal Co, Ltd, were kept in specific pathogen free (SPF) environment for three days before conducting the follow-up experiments. Test and control groups of 1x10^7^ cells were simultaneously injected into the mice by subcutaneous injection. The volume size of the xenograft tumors was measured on the 6th, 12th and 18th days after injection, respectively. Animal imaging was used to observe tumor growth in mice in real time [11].

### Differentially expressed genes (DEG)

Gastric cancer RNA sequencing data were processed by R package limma, pheatmap and ggplot2 for screening differentially expressed genes and presenting the top 50 DEGsin a heat map [12]. The screening criteria were P-value less than 0.05 and |LogFC|≥2.

### WGCNA and identification of key module

Unlike the focus on differentially expressed genes, Weighted Gene Co-expression Network analysis (WGCNA) analyzed the data based on two assumptions: 1 Genes with similar expression patterns may be co-regulated, functionally related or under the same signaling pathway. 2 The genes in the network obey scaleless network distribution [13]. After removing the abnormal samples, the Pearson correlation coefficient between any paired genes was calculated. We then build the weight adjacency matrix by the power function amn = |cmn|β method [14]. A suitable β value is determined to remove weak correlations between genes, and therefore more conducive to building co-expression network. In the next step, we transform the weight adjacency matrix into a topological overlap matrix (TOM) so that we can measure the connectivity of genes in the network. Based on the TOM measurements, average linkage hierarchical clustering is used to classify genes with similar expression profiles with the same module. A minimum size of 50 per group is the criterion for gene dendrograms [13].

### Consensus clustering

After finding key genes by WGCNA and mRNAsi, we applied consensus clustering analysis to divide TCGA patient samples into different subtypes. R package ConsensusClusterPlus completed the above analysis. Cumulative distribution function (CDF) and consensus matrices determine the appropriate number of subgroups [15].

### Functional annotation

Gene ontology (GO) and Kyoto Encyclopedia of Genes and Genomes (KEGG) analyses were applied to characterize the biological function of genes you are interested. And these analyses were carried out by applying these doses, clusterProfiler, org.Hs.eg.db, enrichplot and ggplot2 R packages [14, 16].

### Construction of risk score models

LASSO (least absolute shrinkage and selection operator) regression analysis and Kaplan-Meier survival analysis were used to construct risk score model [11, 17, 18].

### Statistical analysis

Data are presented as mean±standard deviation. A P-value of less than 0.05 was considered significantly different.(*P< 0.05; **P< 0.01; ***P< 0.001;****P< 0.0001).

### Availability of supporting data

The data generated during this study are included in this article and its Supplementary Information files are available from the corresponding author on reasonable request.

## RESULTS

### Detection of differences in mRNAsi and differentially expressed genes in gastric cancer

**Figure 1:**
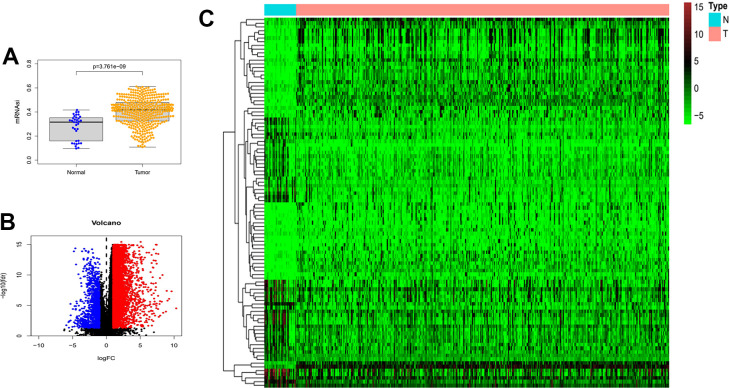
**Differences in mRNAsi and sample gene expression.** (**A**) Differences in mRNAsi between normal and tumor tissues in gastric cancer. (**B**) Volcano map of differentially expressed genes. Green dots represent genes that are down-regulated, red dots represent genes that are up-regulated, and black dots represent no significant change. (**C**) The top 50 differentially expressed genes in GC cancer disease presented as a gene expression heat map. *P*<0.05. GC: gastric cancer.

The mRNAsi is a widely recognized parameter for determining the similarity between tumor cells and normal stem cells. We first explored the differences in mRNAsi in normal and tumor samples of gastric cancer. As shown in Figure 1A, mRNAsi was dramatically different between the two groups, with the tumor group samples possessing much higher mRNAsi values than the normal. Subsequently, we screened differentially expressed genes in TCGA gastric cancer RNA sequencing data. Limma and pheatmap R packages processed the above data and extracted the top 50 DEGs to plot as heat map and volcano map (Figure 1B, 1C). In total, we obtained 6736 differential genes of which 1139 expressed down-regulated genes and 5597 up-regulated genes.

### Identification of mRNAsi-related key genes and their functional annotation

**Figure 2:**
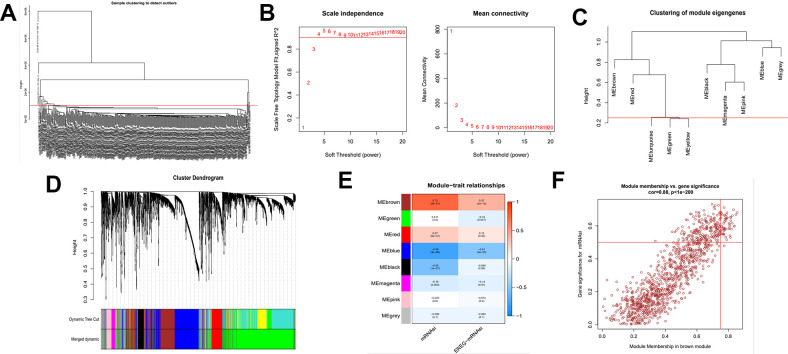
**Identification of cancer stem cell index-related modules by WGCNA.** (**A**) Samples above the red line were removed because they were considered as the deflection of gene expression. (**B**) This represents the correlation coefficient R^2^ and mean connectivity in the scale-free network. (**C**) Calculate similarity between modules and merge modules with high similarity. (**D**) Hierarchical clustering of gene modules. (**E**) Heatmap of the correlationship between gene modules and cancer stemness index. (**F**) Scatter plot of maximum positive correlation with cancer stem cell index (mRNAsi).

The above findings demonstrate that there may be genes that play a critical role in regulating tumor stemness in DEGs. Therefore, we applied WGCNA and mRNAsi to search for these genes more deeply. After DEGs were processed by the WGCNA algorithm, we first removed the samples that did not meet the threshold because t of the deflection of their gene expression (Figure 2A). We then select β=4 (scalefree R^2^=0.9) as a soft threshold to build the scaleless network (Figure 2B). After calculating the similarity between modules, we merged the modules below the red line (Figure 2C) and plotted the gene dendrogram (Figure 2D). A total of 8 modules were obtained and named with different colors. A heat map was plotted to show the relationship between different modules and tumor stem cell index (Figure 2E). Finally, we chose the brown module as the subject for the subsequent study.

**Figure 3:**
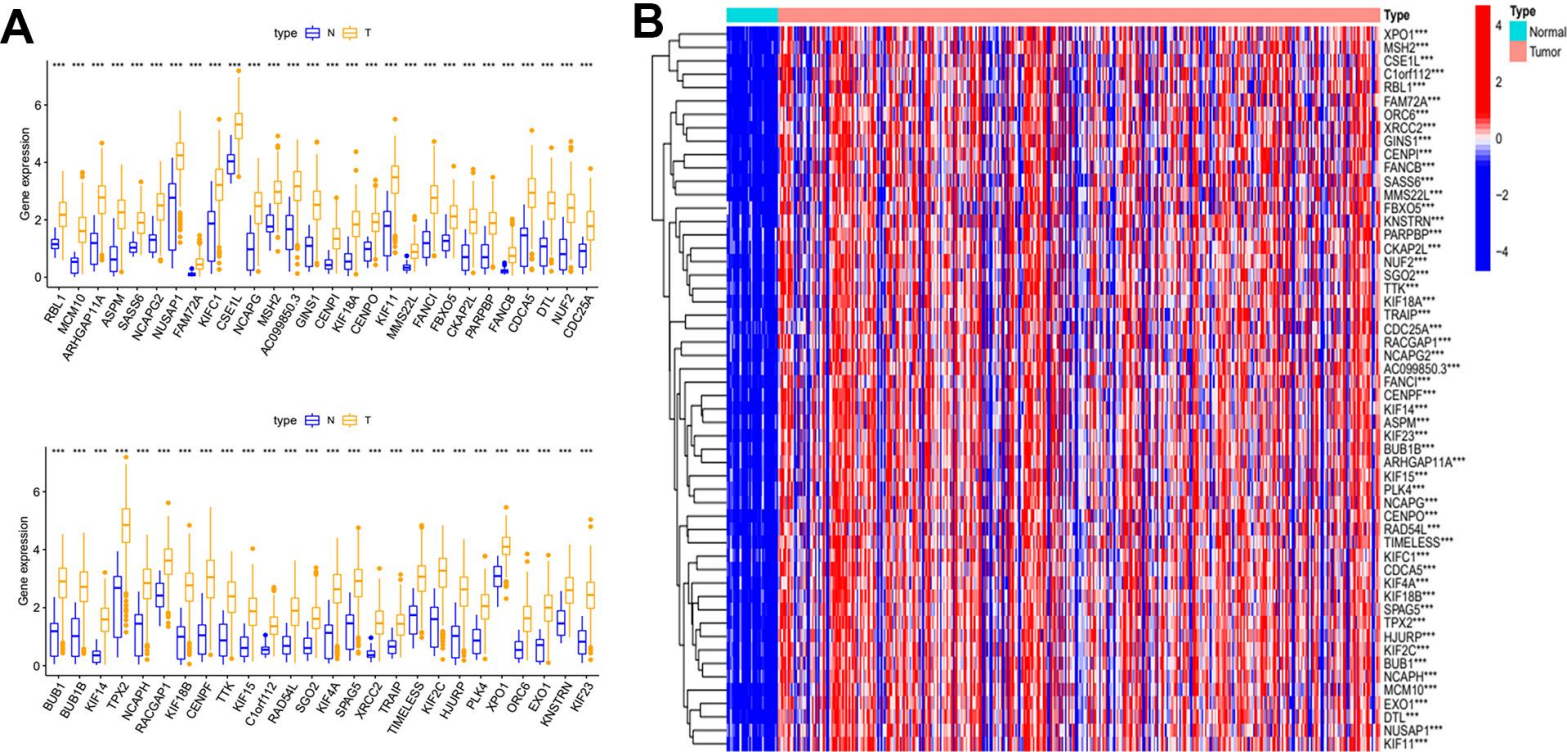
**Differential expression analysis of key genes.** (**A**) Box plot of the difference in expression of key genes between tumour and normal tissue. (**B**) Key genes differential expression heatmap.

**Figure 4:**
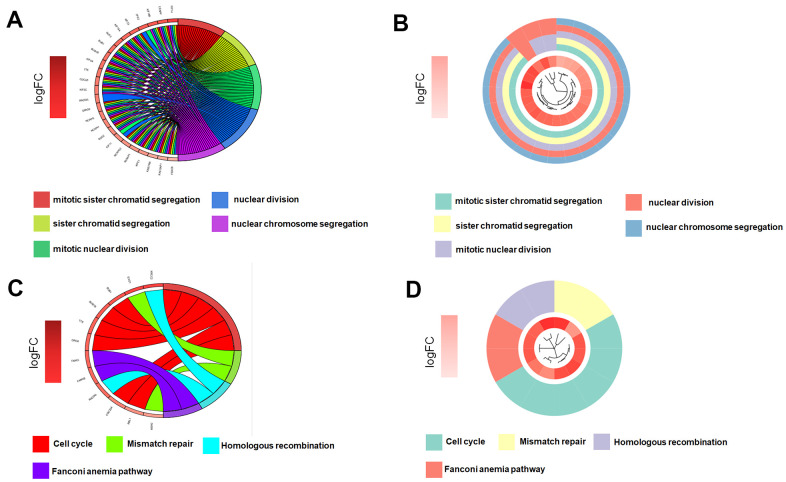
**Key genes function enrichment analysis.** (**A**, **B**) GO enrichment analysis of key genes. (**C**, **D**) KEGG enrichment analysis of key genes.

Gene significance (GS) represents the correlation between the gene and the trait of interest. Module membership (MM) represents the correlation between the module genes and this module. In this study, we set gene significance (GS)>0.5, Module membership (MM)>0.75 as criteria to screen key genes in brown modules (Figure 2F). In total, 54 tumor stemness-related genes were obtained. Firstly, we performed correlation analysis on these 54 genes to demonstrate the accuracy of the above parameter settings (Supplementary Figure 1). And we subsequently extracted the expression data of these genes to map them as box line plots and heat maps (Figure 3A, 3B). Functional enrichment analysis was likewise performed for these genes (Figure 4A, 4B). The results of GO analysis showed that these genes are involved in sister chromatid segregation and cell nuclear division, etc. The results of KEGG are mainly for cell cycle and mismatch repair, etc (Figure 4C, 4D).

### Molecular subtypes of gastric cancer based on mRNAsi-related key genes and identification of key modules

**Figure 5:**
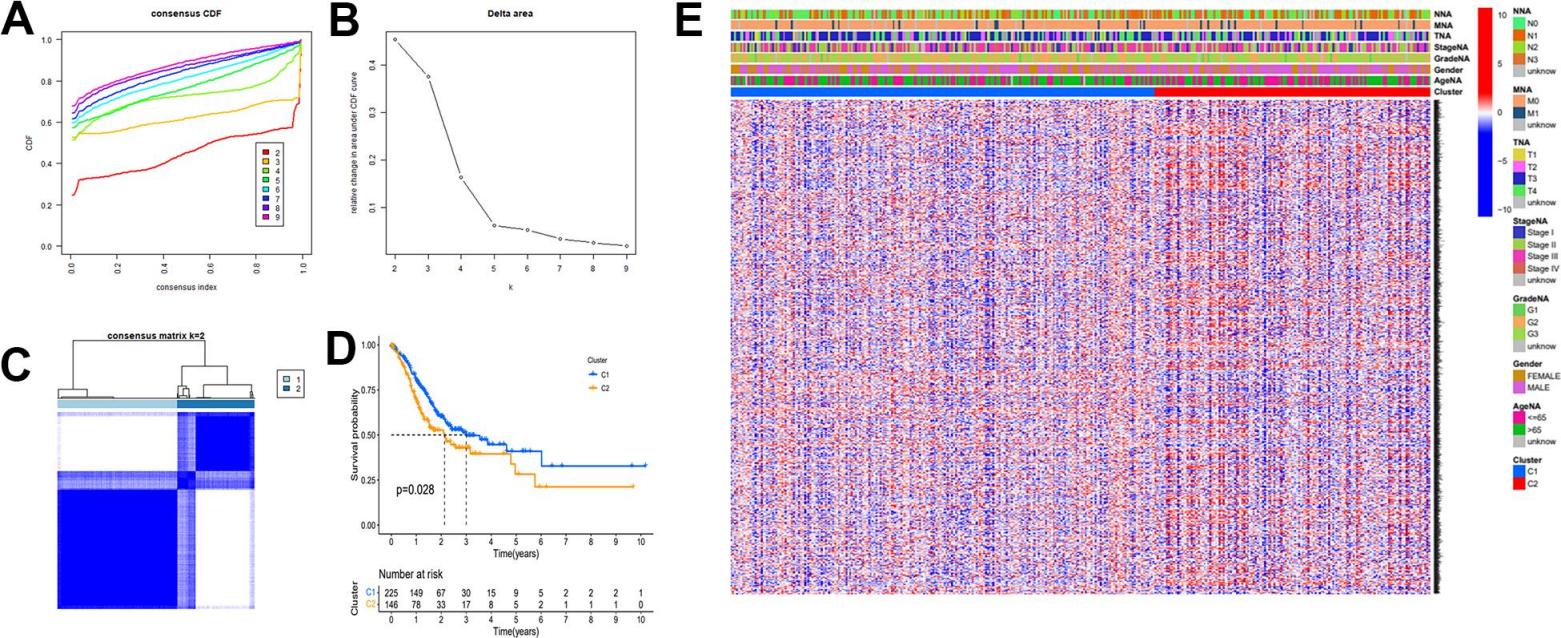
**The mRNAsi-related key genes could classify GC into two groups by consensus clustering of TCGA dataset.** (**A**) Cumulative distribution function (CDF) for k=2 to k=9. (**B**) Relative change in area under the CDF curve according to different k values. (**C**) Consensus clustering matrix of samples from TCGA dataset for k=2. (**D**) Survival analysis of patients in the C1 group and C2 group in TCGA cohort. (**E**) Heatmap of two clusters defined by the expression of mRNAsi-related key genes.

To explore novel investigation objectives and horizons, we conducted a consensus clustering analysis using the obtained tumor stemness-associated key genes. After consensus clustering analysis, the 384 gastric cancer patient samples would be classified into different subtypes. Figure 5A shows the relative change of CDF curve of consensus score from k = 2 to 9. Relative change in area under the CDF curve for k = 2 to 9 (Figure 5B). When k = 2 for consensus clustering, it proves to be the best choice for dividing the patient samples (Figure 5C). Then we performed survival curve analysis between the two groups and their relationship with clinical characteristics. The K-M survival analysis showed that the overall survival rate of the C1 group was higher than that of the C2 group (Figure 5D). Clinical heatmap for two groups was shown in Figure 5E.

**Figure 6:**
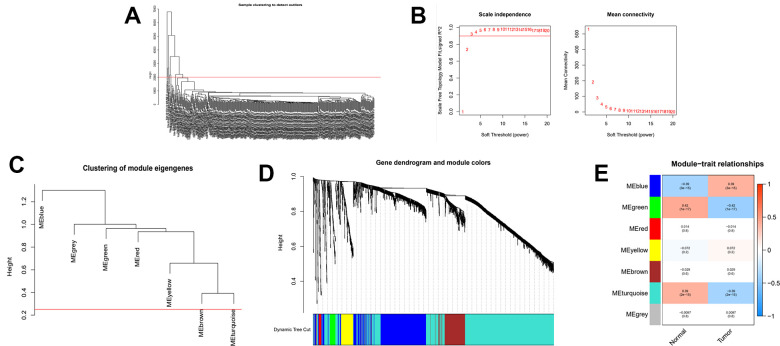
**WGCNA analysis on the consensus clustering samples.** (**A**) Samples above the red line were removed because they were considered as the deflection of gene expression. (**B**) This represents the correlation coefficient R^2^ and mean connectivity in the scale-free network. (**C**) Calculate similarity between modules and merge modules with high similarity. (**D**) Hierarchical clustering of gene modules. (**E**) Heatmap of the correlationship between gene modules and normal or cancer tissue.

In this part of the study, we likewise performed WGCNA analysis on the consensus clustering samples. First filter out the outliers and this time we selected β=4 (scalefree R^2^=0.9) as the parameter to build the network (Figure 6A, 6B). And after merging the high similarity modules (Figure 6C, 6D), the heatmap was obtained (Figure 6E). Finally, we identified the blue module as object due to its maximum positive correlation with tumor to study.

### Establishment and validation of risk prognostic model

**Figure 7:**
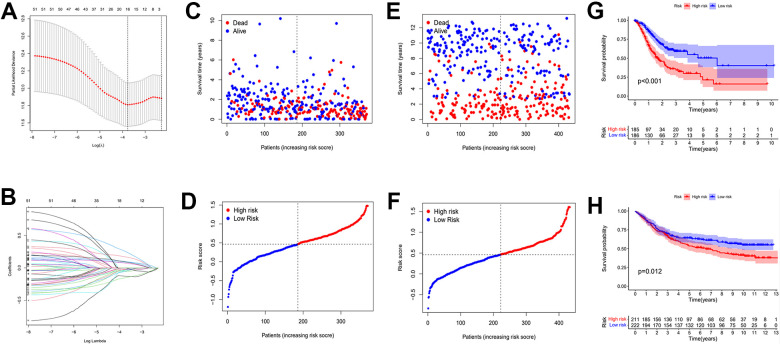
**Establishment of risk prognostic model.** (**A**) Partial likelihood deviance was plotted versus log (Lambda). The vertical dotted line indicates the lambda value with the minimum error and the largest lambda value. (**B**) LASSO coefficient profiles of the genes screening by univariate Cox regression analysis. (**C**, **D**) The distributions of risk scores and OS status in TCGA. (**E**, **F**) The distributions of risk scores and OS status in GEO. (**G**) The patient samples from TCGA were divided into high and low risk groups based on risk score and the OS of the groups were analyzed using Kaplan-Meier. (**H**) OS analysis of high and low risk groups from the GEO samples. Red represents the high risk group and blue represents the low risk group. LASSO: least absolute shrinkage and selection operator. OS: overall survival.

A total of 621 genes were obtained. To investigate the prognostic role of these genes in gastric cancer, a risk prognostic model was constructed. We first perform initial screening and obtained 51 genes (Supplementary Figure 2 and Table 1). We then applied these 51 genes to the LASSO regression algorithm to construct a risk prognostic model (Figure 7A). As a result, we obtained a 16-gene risk model. Six of these genes were positively associated with the overall survival of the sample and ten were negatively associated (Supplementary Figure 3). We applied the coefficient of each risk gene to calculate the risk score for every gastric cancer patient sample.

**Table 1:** Results of the consensus clustering analysis key genes in the univariate Cox regression analysis.

**Univariate analysis**				
**ID**	**HR**	**HR.95L**	**HR.95H**	**Pvalue**
TFDP1	0.735124	0.55663	0.970856	**0.03013**
LMNB2	0.719488	0.576068	0.898614	**0.003703**
FEN1	0.7427	0.5878	0.93842	**0.012684**
GRB14	1.327029	1.047293	1.681484	**0.019153**
SAC3D1	0.733009	0.57856	0.928689	**0.010089**
CHEK1	0.797379	0.637004	0.998131	**0.048122**
DNAJC9	0.660751	0.473493	0.922065	**0.014802**
MASTL	0.7034	0.511674	0.966967	**0.030247**
KIF24	0.605495	0.42476	0.863132	**0.005542**
UPK1B	1.242177	1.086956	1.419564	**0.001451**
KIF15	0.803923	0.654898	0.986858	**0.03694**
HBB	1.148409	1.027815	1.283152	**0.0145**
CHAF1A	0.645577	0.488588	0.85301	**0.002082**
CKAP2	0.796454	0.634945	0.999046	**0.049042**
UHRF1	0.759047	0.618619	0.931354	**0.008259**
THOP1	0.701354	0.538906	0.912771	**0.008317**
BRIP1	0.612228	0.409319	0.915724	**0.016915**
MTBP	0.692778	0.489908	0.979657	**0.037874**
INCENP	0.615693	0.458523	0.826738	**0.001259**
BST1	1.477932	1.116774	1.955887	**0.006285**
TTF2	0.677727	0.500876	0.91702	**0.011687**
GINS4	0.722585	0.546858	0.95478	**0.022286**
GPSM2	0.686969	0.525703	0.897706	**0.005951**
E2F2	0.74841	0.618022	0.906307	**0.003005**
ZNF367	0.643423	0.459573	0.900821	**0.010219**
ASF1B	0.813848	0.683304	0.969331	**0.020935**
USP1	0.635607	0.452134	0.893531	**0.009114**
ADH4	1.149445	1.03336	1.27857	**0.010344**
EZH2	0.741219	0.580489	0.946453	**0.016339**
GTPBP3	0.628953	0.427261	0.925856	**0.01875**
TMEM201	0.668543	0.465236	0.960696	**0.029501**
GAD1	0.762423	0.615481	0.944445	**0.01302**
POLQ	0.774836	0.624203	0.961821	**0.020729**
PGAM5	0.733225	0.552097	0.973775	**0.03207**
L2HGDH	0.708646	0.504273	0.995849	**0.047264**
RAD54L	0.760861	0.605462	0.956145	**0.019043**
ZNF74	0.664294	0.450144	0.980325	**0.039394**
PKMYT1	0.797294	0.646888	0.98267	**0.033681**
CLSPN	0.708654	0.518482	0.968579	**0.030756**
DCLRE1B	0.613154	0.398937	0.9424	**0.025715**
VSNL1	0.869914	0.764511	0.98985	**0.034449**
KIF18B	0.747912	0.568393	0.984129	**0.03806**
DNMT1	0.692752	0.506058	0.948322	**0.021955**
SPC24	0.786286	0.625723	0.98805	**0.039101**
RNF43	0.778565	0.659917	0.918544	**0.003005**
SLC5A6	0.788416	0.624576	0.995235	**0.045484**
SASS6	0.670237	0.465455	0.965116	**0.03149**
ERCC6L	0.679181	0.49137	0.938775	**0.019154**
MAGE-A3	1.106608	1.001073	1.223269	**0.047599**
CDC25A	0.775959	0.621335	0.969063	**0.025278**
SMC1A	0.647933	0.456543	0.919556	**0.015121**

Bold words mean P value is less than 0.05; HR, hazard ratio; L, low; H, high.

The calculation formula is as follows. :(expression of RNF43 x -0.155+expression of INCENP x -0.127+expression of KIF24 x -0.056+expression of PGAM5 x -0.055+expression of SASS6 x -0.04+expression of SAC3D1 x -0.037+expression of TTF2 x -0.034+expression of MASTL x -0.023+expression of E2F2 x -0.021+expression of GAD1 x -0.018+expression of HBB x 0.07+expression of UPK1B x 0.08+expression of MAGE-A3 x 0.09+expression of ADH4 x 0.1+expression of BST1 x 0.17+expression of GRB14 x 0.25). The TCGA cohorts and the externally validated cohorts (GSE88437) can be divided into high and low risk groups. The distribution of patient survival status and risk scores from the TCGA database and external validation database were presented in Figure 7C–7F. The scatter plot show that as the patient risk score increases the proportion of patient deaths also increases. Kaplan-Meier survival analysis displayed that overall survival of the high-risk group compared to that of the low-risk group was lower.

**Figure 8:**
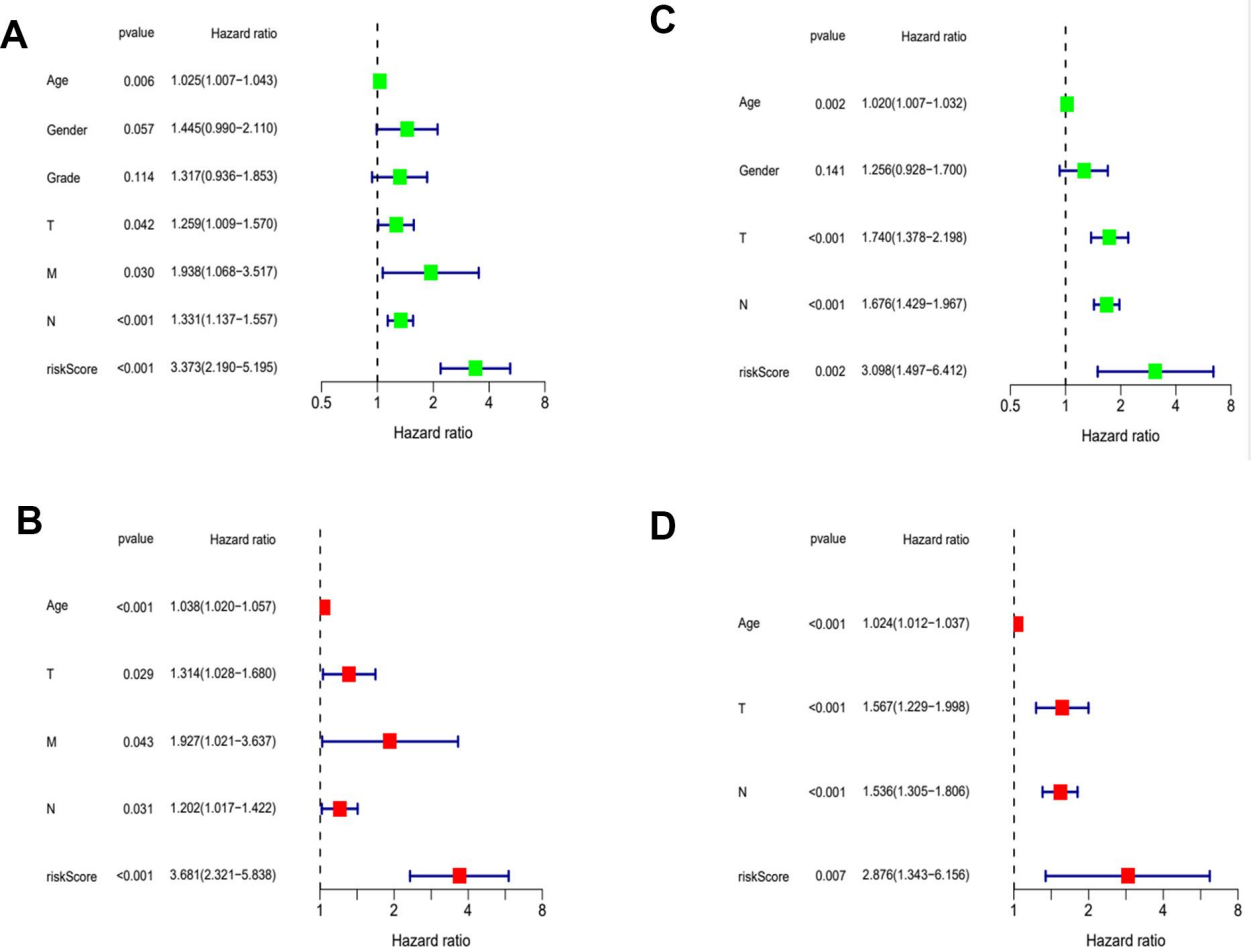
**Evaluation of risk model.** (**A**) Univariate Cox analysis of risk score and clinical characteristics in TCGA. (**B**) Univariate Cox analysis in GEO. (**C**) Multivariate Cox analysis of risk score and clinical characteristics in TCGA. (**D**) Multivariate Cox analysis in GEO.

**Figure 9:**
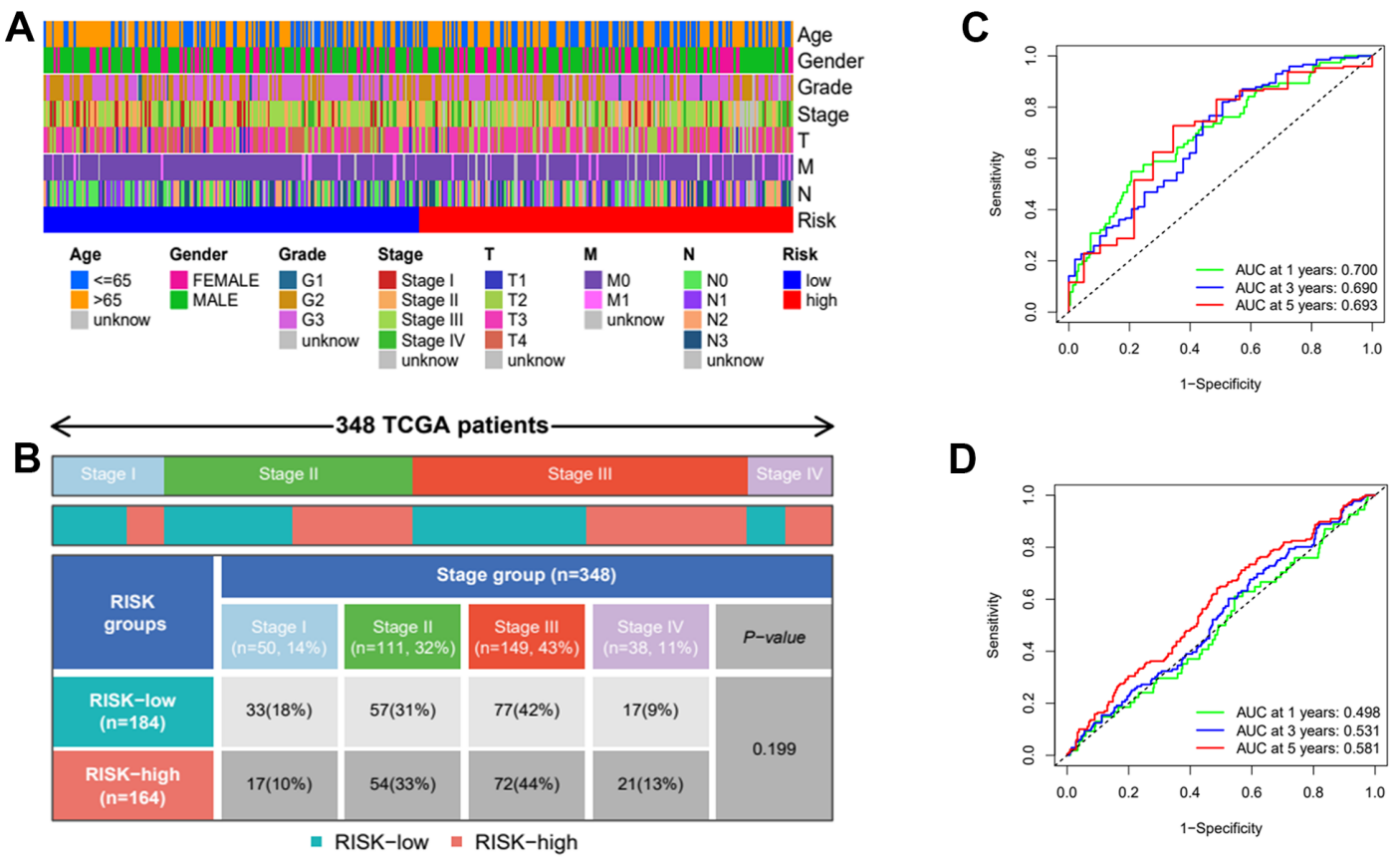
**Clinical heatmap of risk scores and time-dependent ROC curve analysis.** (**A**) Heatmap of risk scores under different clinical characteristics. (**B**) Distribution of high and low risk groups under different clinicopathological stages. (**C**) ROC curve analysis in TCGA. (**D**) ROC curve analysis in GEO.

Then, we applied Cox regression analyses to evaluate the risk model. The results in Figure 8A, 8B and Table 2 were from the TCGA database, which demonstrates that age, TNM, and risk score were all significantly associated with OS. And results in Figure 8C, 8D and Table 3 were from the external validation dataset and the obtained results again indicated that the risk score is significantly associated with OS. Clinical heat maps of risk scores and other clinical characteristics are shown in Figure 9A, 9B. Then, we evaluated this model with a time-dependent ROC curve. The AUC values for 1, 2, and 3 years in the TCGA cohort were 0.7, 0.69, and 0.693, respectively (Figure 9C). The AUC values for 1, 2, and 3 years in the external validation cohort were 0.498, 0.531, and 0.581, respectively (Figure 9D).

**Table 2:** Results of the risk score and clinical characteristics in the univariate and multivariate Cox regression analysis.

**Univariate analysis**					**Multivariate analysis**			
**Parameter**	**HR**	**HR.95L**	**HR.95H**	**Pvalue**	**HR**	**HR.95L**	**HR.95H**	**Pvalue**
Age	1.024932	1.007256	1.042918	0.005528	1.038105	1.019845	1.056692	3.62E-05
Gender	1.445034	0.989755	2.109736	0.056568				
Grade	1.31705	0.936361	1.852513	0.113605				
T	1.258574	1.008851	1.570111	0.041545	1.314296	1.028257	1.679904	0.029074
M	1.937808	1.067767	3.516777	0.029585	1.926676	1.02064	3.637013	0.043075
N	1.33078	1.137148	1.557382	0.000368	1.202353	1.016749	1.421838	0.03123
riskScore	3.372834	2.189648	5.195361	3.47E-08	3.681065	2.320963	5.838198	3.06E-08

Bold words mean P value is less than 0.05; HR, hazard ratio; L, low; H, high.

**Table 3:** Results of the risk score and clinical characteristics in the univariate and multivariate Cox regression analysis.

**Univariate analysis**					**Multivariate analysis**			
**Parameter**	**HR**	**HR.95L**	**HR.95H**	**Pvalue**	**HR**	**HR.95L**	**HR.95H**	**Pvalue**
Age	1.019603	1.006985	1.032379	0.002246	1.024352	1.011999	1.036856	0.000102
Gender	1.255635	0.927537	1.699792	0.140707				
T	1.740416	1.37793	2.19826	3.31E-06	1.566935	1.228577	1.998479	0.000296
N	1.676345	1.428609	1.967042	2.42E-10	1.535546	1.305359	1.806324	2.27E-07
riskScore	3.098135	1.49706	6.411528	0.002309	2.875592	1.343156	6.156421	0.006537

Bold words mean P value is less than 0.05; HR, hazard ratio; L, low; H, high.

### MAGE-A3 possess the property to regulate tumor stemness and proliferation through PI3K/AKT signaling pathway

**Figure 10:**
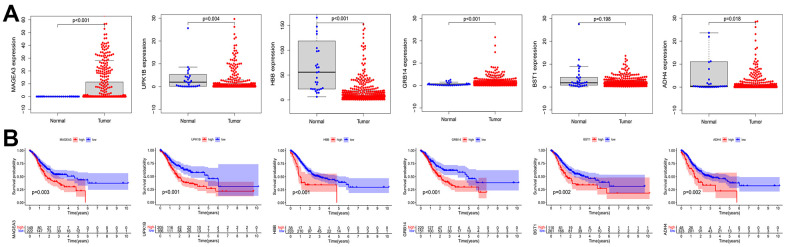
**Expression and prognostic role of OS positive-related genes.** (**A**) Analysis of expression differences of OS positive-related genes. (**B**) Kaplan-Meier analysis of OS positive-related genes. OS: overall survival.

**Figure 11:**
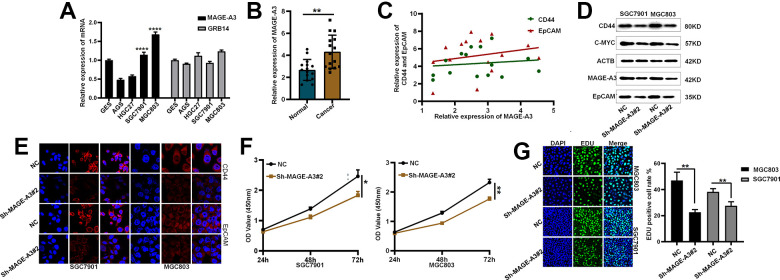
**Validation of MAGE-A3’s regulation of tumor stemness and proliferative capacity *in vitro*.** (**A**) Application of QPCR to compare MAGE-A3 and GRB14 mRNA expression in tumour cells and normal epithelial cells. (**B**) The expression of MAGE-A3 in cancer and adjacent tissues was detected by QPCR. (**C**) Relationship between the expression of CD44 and EpCAM and the expression of MAGE-A3. (**D**, **E**) Validation of protein expression levels of cancer stem cell biomarkers by western blot and immunofluorescence. (**F**, **G**) The effect of knocking down MAGE-A3 on cell proliferation ability was examined by CCK-8, EDU. (*P< 0.05; **P< 0.01; ***P< 0.001;****P< 0.0001).

After considering the expression of these OS positive-related risk genes and role of prognosis, we selected MAGE-A3 and GRB14 as the subsequent study subjects (Figure 10). As shown in Figure 11A, MAGE-A3 was significantly highly expressed in gastric cancer cell lines MGC803 and SGC7901, while the difference in GRB14 expression was not significant. Subsequently, we detected the expression of MAGE-A3 in cancer and normal tissues from gastric cancer samples, and the results showed that MAGE-A3 is highly expressed in tumor tissues (Figure 11B). In order to verify the relationship between MAGE-A3 and tumor stemness regulation, we also detected the expression of tumor stem cell markers CD44 and EpCAM in these tissues. As shown in the Figure 11C, the expression of CD44 and EpCAM increased with the expression of MAGE-A3. Therefore, MAGE-A3 was selected as the final target of the study. We constructed MAGE-A3 knockdown stable transgenic cell in gastric cancer cell lines. Subsequently, cancer stem cell biomarkers were detected after MAGE-A3 knockdown. As shown in Figure 11D, 11E, knockdown of MAGE-A3 resulted in a significant decrease in protein expression of these biomarkers. We also examined the effect of MAGE-A3 on cell proliferation. The thymidine analog EDU can be incorporated into newly synthesized DNA in place of thymidine during the S phase of the cell cycle. The results of the EDU experiments were similar to those described above. Knockdown of MAGE-A3 reduced the ability of the cells to synthesize DNA (Figure 11F). The results of CCK-8 experiments showed that the knockout of MAGE-A3 decreased the proliferation ability of SGC7901 cells by 25.6% and MGC803 by 24.1% (Figure 11G).

**Figure 12:**
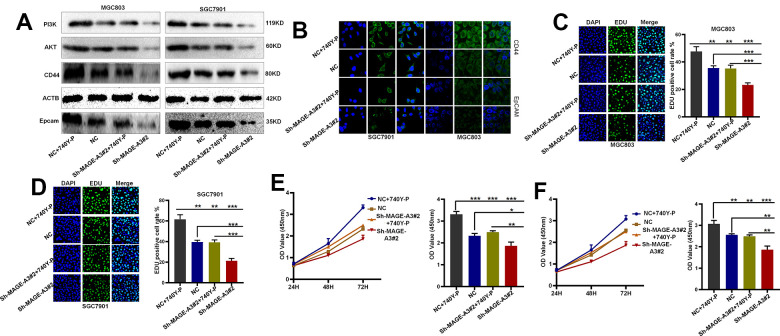
**MAGE-A3 regulates tumour stemness and proliferation through the PI3K/AKT pathway.** (**A**, **B**) Western blot and cellular immunofluorescence techniques were used to detect the expression of PI3K/AKT and tumour stem cell protein biomarkers under different grouping treatments. (**C**, **D**) EDU assays were used to detect the proliferation ability of cells under different grouping treatments. (**E**, **F**) CCK-8 assays were used to detect the proliferation ability of cells under different grouping treatments.

**Figure 13:**
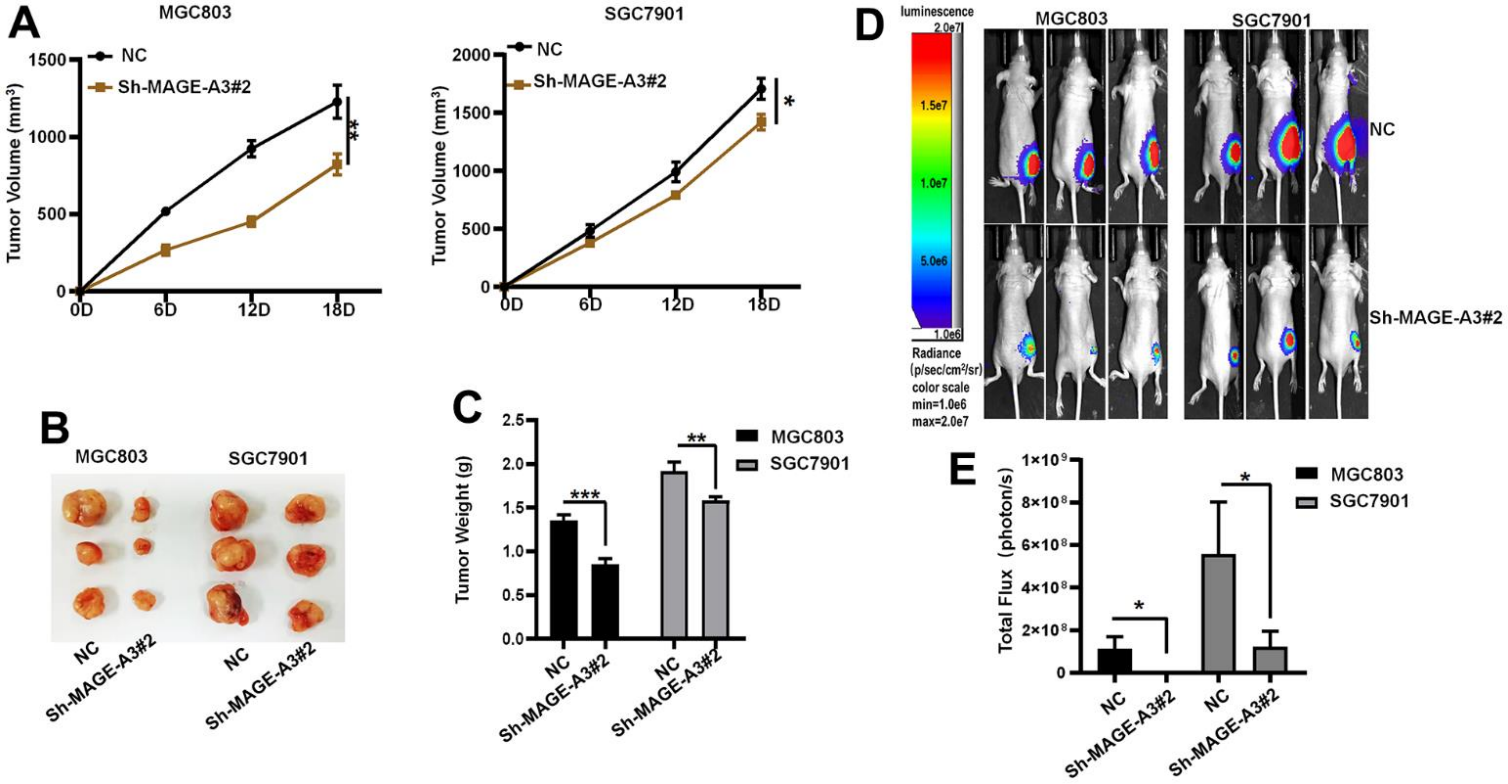
**Verifying the ability of MAGE-A3 to regulate tumors *in vivo*.** (**A**) Tumor volume growth curves of control group and knockdown group. (**B**) Xenograft tumors of sacrificed mice at the experimental endpoint. (**C**) Tumor weights in control and knockdown groups. (**D**, **E**) Animal imaging technology to detect differences between control and knockdown groups. (*P< 0.05; **P< 0.01; ***P< 0.001;****P< 0.0001).

To verify through which signaling pathway MAGE-A3 exerts its ability to regulate tumor stemness and proliferation. We applied Western Blot technique to detect PI3K/AKT signaling pathway and applied 740Y-P, an activator of this signaling pathway, to MAGE-A3 knockdown cells. The results as shown in Figure 12A showed that the expression of PI3K and AKT decreased significantly after knockdown of MAGE-A3, but their expression rebounded significantly after treatment with activator 740Y-P. Meanwhile, the expression of CD44 and EpCAM varied with the expression of PI3K and AKT (Figure 12B). The results of CCK-8 and EDU experiments also showed that the proliferation ability of cells significantly increased when 740Y-P was added (Figure 12C–12F). These results demonstrate that MAGE-A3 may achieve its role in regulating tumour stemness and proliferation through the PI3K/AKT signalling pathway. Finally, we performed *in vivo* experiments that were used to verify the effect of MAGE-A3 on tumor growth. The constructed knockdown and control cells were injected simultaneously into different groups of nude mice subcutaneously. The volume size of the xenograft tumors in mice was measured at different time points after injection, respectively. Finally, at day 18, The mice were imaged and then sacrificed to remove the tumor and measure their weight. As shown in Figure 13A, 13B, the mean reduction in the knockdown group compared to the control group was 405 and 285 cubic millimeters, respectively. In terms of tumor weight, the knockdown group injected with MGC803 cells was reduced by 0.5 g and SGC7901 by 0.334 g (Figure 13C). The results obtained from the animal imaging technique showed that the bioluminescence of knockdown groups significantly lower than the control groups (Figure 13D, 13E).

## DISCUSSION

Despite the great contribution of new therapies to the treatment of cancer, some patients still own poor outcomes, prompting us to search for potential molecular mechanisms to address this issue. Tumor heterogeneity has always been a challenge for oncology treatment. The study of tumor stem cells, a special class of stem cell-like tumor cells, has been recently springing up vigorously. With the property of stem cell-like and resistant to chemotherapy and radiotherapy, tumor stem cells are involved in tumor multiple processes such as tumorigenesis, progression, metastasis, and recurrence after therapy [19–22]. Therefore, this study focuses on the molecular mechanisms regulating the properties of cancer stem cells.

In 2018 Malta and his colleagues introduced the concept of cancer stemness index, a concept used to describe the degree of tumor differentiation. The cancer stemness index was rapidly applied to study cancer stem cells in different cancer, such as lung adenocarcinoma, breast cancer, pancreatic cancer, etc. [23–27]. In this study, we first applied WGCNA and mRNAsi to construct a co-expression network based on differentially expressed genes to obtain modules with different degrees of correlation with mRNAsi. We select the brown module and then determine the stemness-related key genes. The results of functional annotation of key genes show that they are mainly responsible for cell cycle, chromosome segregation, etc. [24, 28]. This finding is consistent with the results of previous studies. Compared with the early research, our study performed consensus clustering analysis on gastric cancer samples based on stemness -related key genes and identified two molecular subtypes of gastric cancer (C1 and C2 groups). Then, we again construct the co-expression network after consensus clustering and select the blue module as the subject study target.

We then construct the stemness subtype-related risk prognostic model with the LASSO regression analysis after Univariate COX regression analysis preliminarily screening the genes within the blue module. The results of Kaplan Meier analysis showed that the OS of patients in the high-risk group was significantly lower than that in the low-risk group, and this result was validated by the GES88437 dataset. COX regression analyses demonstrate that this risk-prognostic model can be used as an independent prognostic factor to predict the outcomes of gastric cancer patients, providing a new basis and possibility for precise treatment and management of patients.

This risk prognostic model contains 16 risk genes, 6 of which are positively associated with OS. UPK1B is a member of the four-transmembrane superfamily, and most members of this family are characterized by four hydrophobic structural domains. This protein is found in asymmetric unit membranes and can interact with other family members to form complexes. And this complex may function in normal bladder epithelial physiology to regulate membrane permeability of superficial umbrella cells or stabilize the apical membrane through AUM/cytoskeleton interactions [29]. High expression of UPK1B in clinical samples of bladder cancer was highly correlated with lymph node metastasis, distant metastasis and advanced stage of tumor. And *in vitro* experiments, UPK1B knockdown affects cell proliferation, migration and invasion through Wnt/β-catenin signaling pathway [30]. The melanoma-associated antigen A (MAGE-A) subfamily is one of the most thoroughly studied members of the cancer/testis antigens (CTA) family, whose expression is characterized by specific expression in various tumor tissues but not in normal tissues, except for germline cells [31]. Therefore, based on their expression characteristics, members of the MAGE-A subfamily have been developed as targets for immunotherapy such as vaccines and CAR-T cells [32]. MAGE-A3 plays prognostic role in many cancers and promotes cancer proliferation, migration, invasion and drug resistance [33–35]. In hepatocellular carcinoma, MAGE-A3 is highly expressed in cancerous tissues and is associated with poor patient prognosis. Knockdown of this protein, which is regulated by LINC01234 and miR-31-5p, affects tumor proliferation, invasion and cisplatin-induced apoptosis [36]. In line with previous findings, we also found that MAGE-A3 is associated with poor prognosis in gastric cancer patients and plays an integral role in the progression of the tumor. However, it is noteworthy that our study appears to be the first to suggest that MAGE-A3 is involved in the regulation of tumour stemness after constructing a stemness subtype- related risk prognostic model. And subsequent *in vitro* and *in vivo* experiments provided favorable evidences support for this finding. And our experimental results observed that MAGE-A3 may regulate tumor stemness and proliferation by PI3K/AKT signaling pathway. And in recent years, this signaling pathway has been frequently reported to be involved in the regulation of tumor stemness in many tumors [37–41] However, our study also has some limitations. First, we only conducted studies at the mRNA level, and the exploration at the protein level is relatively limited. Second, we have not yet investigated the mechanism of how MAGE-A3 specifically regulates tumor stemness. And this will also be the focus of our next work. Further studies on the molecular mechanisms of MAGE-A3 will deepen our understanding of the pathological mechanisms of gastric cancer, and this will provide new directions for the treatment.

In summary, we have obtained a 16-gene risk-prognosis model based on WGCNA, consensus clustering analysis and LASSO analysis. This model was validated and can be used to predict disease progression or treatment progression in patients. Finally, we screened these 16 genes and found that MAGE-A3 possesses the ability to regulate tumour stemness, and these findings suggest that MAGE-A3 may be an effective potential target for gastric cancer cure.

## Supplementary Material

Supplementary Figures
