# Supplementary material for: MAGE-A3 regulates tumor stemness in gastric cancer through the PI3K/AKT pathway
Source: Aging (Albany NY). 2022 Nov 8;14(23):9579–98. doi: 10.18632/aging.204373 (PMC9792200; doi:10.18632/aging.204373)
Supplement: Supplementary Figures [file aging-14-204373-s001.pdf]

SUPPLEMENTARY FIGURES

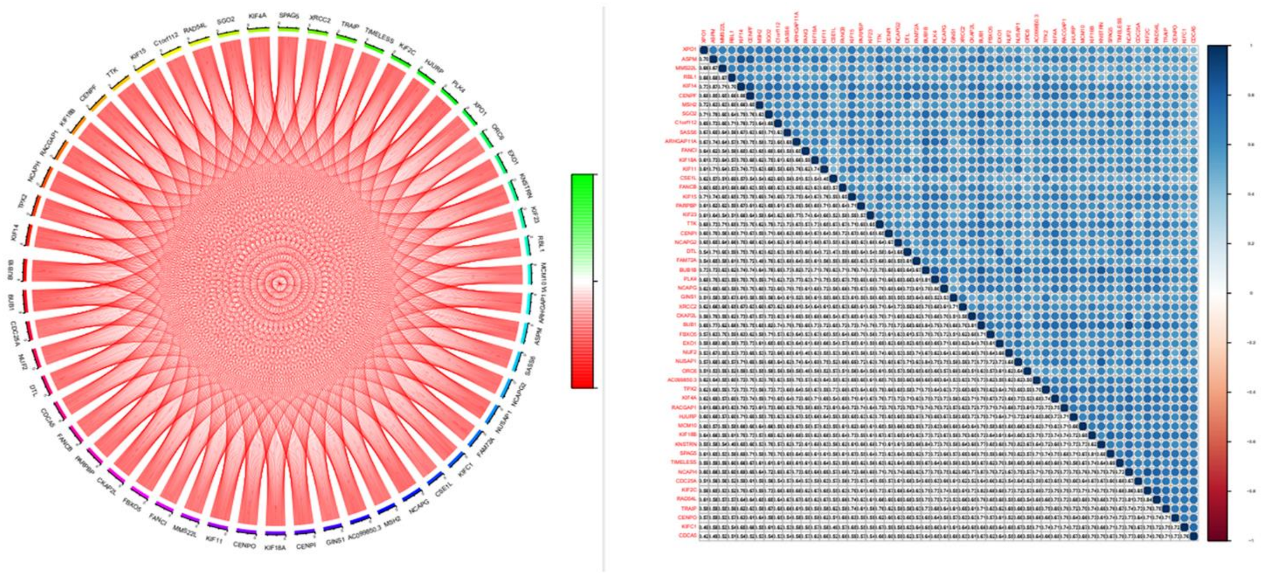

Supplementary Figure 1. Correlation between key genes.

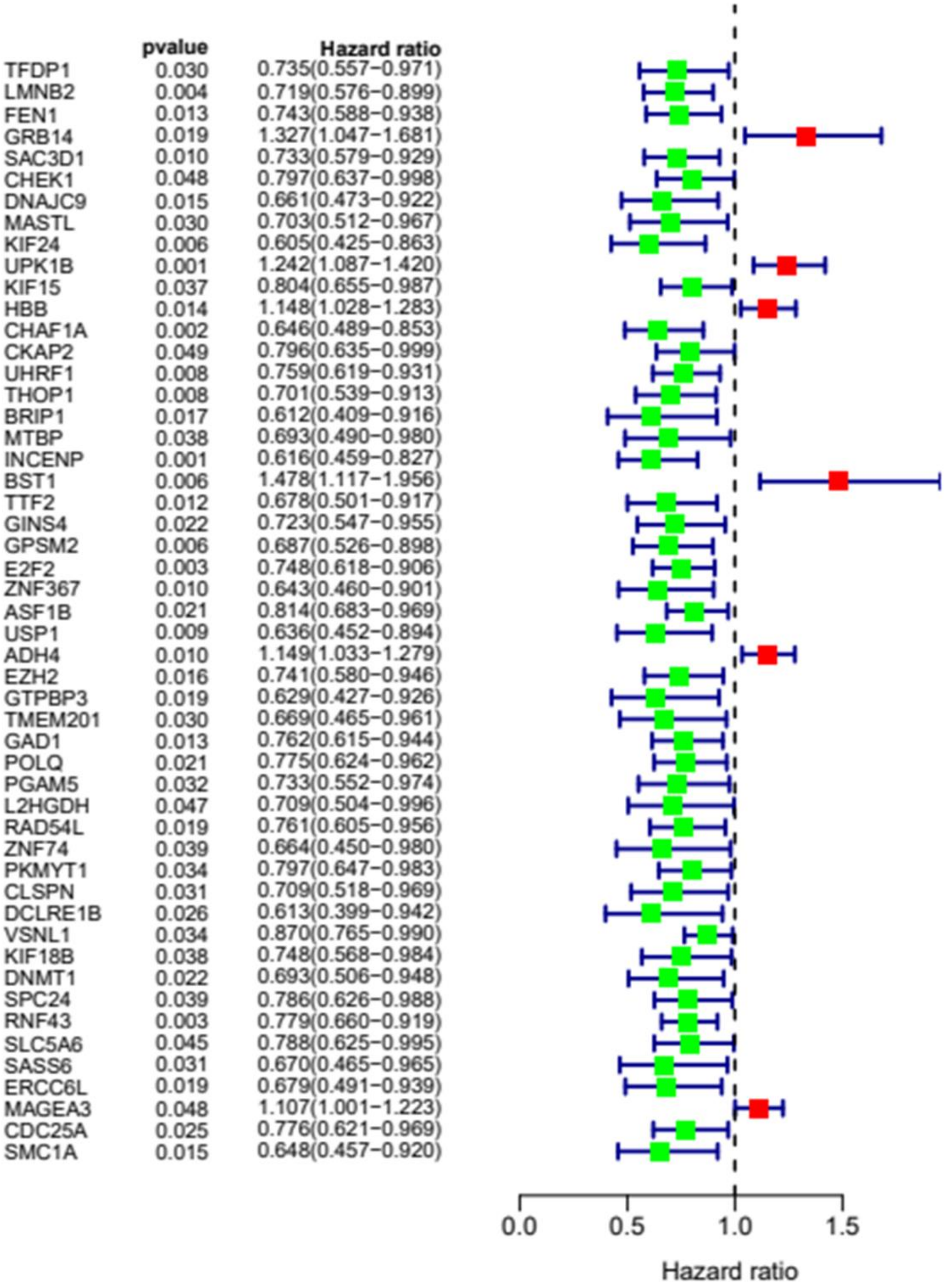

Supplementary Figure 2. Univariate Cox analysis of the prognostic value of key genes.

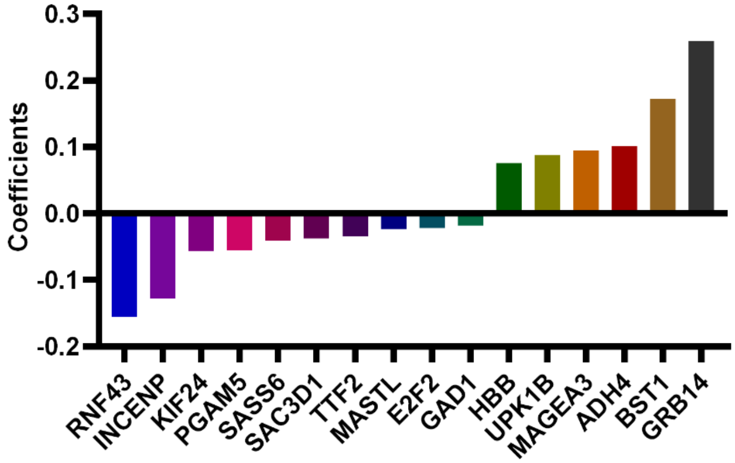

Supplementary Figure 3. The 16 genes were selected by least absolute shrinkage and selection operator. (LASSO) Cox analysis in TCGA dataset and histogram of coefficient.
